# Effect of Longer Family Meals on Children’s Fruit and Vegetable Intake: A Randomized Clinical Trial

**DOI:** 10.1001/jamanetworkopen.2023.6331

**Published:** 2023-04-03

**Authors:** Mattea Dallacker, Vanessa Knobl, Ralph Hertwig, Jutta Mata

**Affiliations:** 1 Center for Adaptive Rationality, Max Planck Institute for Human Development, Berlin, Germany; 2 Division of Health Psychology, University of Mannheim, Mannheim, Germany

## Abstract

**Importance:**

Family meals are a formative learning environment that shapes children’s food choices and preferences. As such, they are an ideal setting for efforts to improve children’s nutritional health.

**Objective:**

To examine the effect of extending the duration of family meals on the fruit and vegetable intake in children.

**Design, Setting, and Participants:**

This randomized clinical trial used a within-dyad manipulation design and was conducted from November 8, 2016, to May 5, 2017, in a family meal laboratory in Berlin, Germany. Included in the trial were children aged 6 to 11 years who did not follow a special diet or have food allergies and adult parents who served as the nutritional gatekeeper in the household (ie, the family member responsible for at least half of the food planning and preparation). All participants underwent 2 conditions: control, defined as regular family mealtime duration, and intervention, defined as 50% longer mealtime duration (10 minutes longer on average). Participants were randomized to the condition they would complete first. Statistical analyses of the full sample were conducted between June 2 and October 30, 2022.

**Interventions:**

Participants had 2 free evening meals under different conditions. In the control or regular condition, each dyad ate in the same amount of time as their reported regular mealtime duration. In the intervention or longer condition, each dyad had 50% more time to eat than their reported regular mealtime duration.

**Main Outcomes and Measures:**

The primary outcome was the number of pieces of fruits and vegetables eaten by the child during a meal.

**Results:**

A total of 50 parent-child dyads participated in the trial. Parents had a mean (range) age of 43 (28-55 years) years and were predominantly mothers (36 [72%]). Children had a mean (range) age of 8 (6-11) years and included an equal number of girls and boys (25 [50%]). Children ate significantly more pieces of fruits (*t*_49_ = 2.36, *P* = .01; mean difference [MD], 3.32 [95% CI, 0.96 to ∞]; Cohen *d* = 0.33) and vegetables (*t*_49_ = 3.66, *P* < .001; MD, 4.05 [95% CI, 2.19 to ∞]; Cohen *d* = 0.52) in the longer condition than in the regular mealtime duration condition. Consumption of bread and cold cuts did not significantly differ between conditions. The children’s eating rate (bites per minute over the regular mealtime duration) was significantly lower in the longer than in the regular condition (*t*_49_ = −7.60, *P* < .001; MD, −0.72 [95% CI, −0.56 to ∞]; Cohen *d* = 1.08). Children reported significantly higher satiety after the longer condition (*V* = 36.5, *P* < .001).

**Conclusions and Relevance:**

Results of this randomized clinical trial suggest that the simple, low-threshold intervention of increasing family mealtime duration by approximately 10 minutes can improve the quality of children’s diet and eating behavior. The findings underscore the potential for such an intervention to improve public health.

**Trial Registration:**

ClinicalTrials.gov Identifier: NCT03127579

## Key Points

**Question:**

How does increased family mealtime duration affect children’s fruit and vegetable intake?

**Findings:**

In this randomized clinical trial of 50 parent-child dyads, children aged 6 to 11 years ate significantly more fruits and vegetables when family meals lasted approximately 10 minutes longer. Intake of other foods offered did not increase.

**Meaning:**

Findings of this trial indicate that increasing family mealtime duration is a simple, inexpensive, and low-threshold intervention that can significantly improve children’s diets.

## Teaser

This randomized clinical trial assesses the effect of family mealtime duration on fruit and vegetable intake among children.

## Introduction

Low fruit and vegetable intake increases the risk for chronic noncommunicable diseases.^1,2^ Yet children worldwide eat considerably less fruits and vegetables than the recommended amount.^3^ Family meals are central to children’s nutrition, with about two-thirds of their calorie intake coming from food prepared at home^4^ and most meals being eaten in the family setting.^5^ Family meals thus serve as a formative learning environment that shapes the food choices and preferences of children.^6^

A meta-analysis of observational studies identified several components of family mealtimes that were associated with better nutritional health in children.^7^ A longer mealtime duration was the most beneficial. This finding may seem counterintuitive considering that longer mealtimes were reported to be associated with greater food intake.^8^ However, many of these studies focused on social occasions with an overabundance of festive foods^9^ or longer exposure to food^10^ and on adults rather than children. Everyday family meals, in contrast, are embedded in daily routines^5^ and typically involve more fruits and vegetables compared with meals eaten outside the home.^11,12,13^ As such, increasing the duration of everyday family meals may increase children’s exposure to, and potentially consumption of, healthy foods. Furthermore, eating as a family may have additional (indirect) effects on children’s eating behavior, including a positive mealtime atmosphere, which in turn is associated with better nutrition quality.^7^ It could also prompt children to eat at a slower pace, which can enhance satiety (ie, feeling full) and reduce food intake.^14,15^

In this randomized clinical trial, we aimed to examine the effect of extending the duration of family meals on the fruit and vegetable intake in children. In terms of this primary outcome, we hypothesized that children eat more fruits and more vegetables when the regular family mealtime duration is extended. We also explored when additional fruits and vegetables were eaten and whether longer meals led to increased consumption of other foods and beverages. In terms of secondary outcomes, we hypothesized that longer family meals facilitate a more positive mealtime atmosphere, decrease eating rates, and increase satiety that, in turn, will lead to lower intake of dessert.

## Methods

From November 8, 2016, to May 5, 2017, we conducted a within-dyad randomized clinical trial involving parent-child dyads, which consisted of 1 parent and 1 child aged 6 to 11 years. The Max Planck Institute for Human Development Ethics Committee approved the trial protocol (Supplement 1). Parents provided written informed consent, and children provided oral consent. We followed the Consolidated Standards of Reporting Trials (CONSORT) reporting guideline.

### Participants

**Figure 1.**
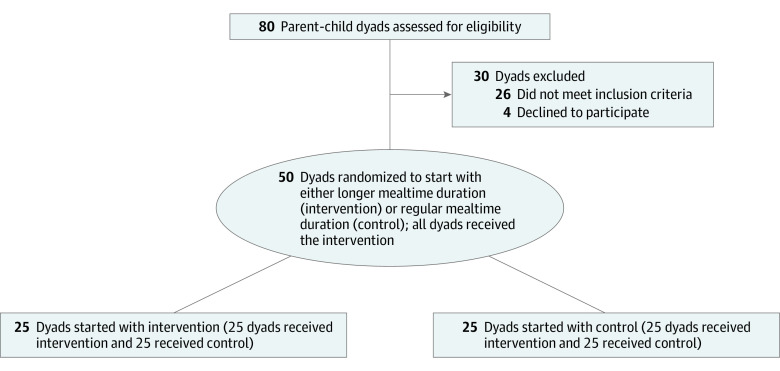
Flow Diagram

Eighty parent-child dyads were recruited to participate. Of these dyads, 26 did not meet the inclusion criteria and 4 declined to participate (Figure 1). Included in the trial were children aged 6 to 11 years who did not follow a special diet or have food allergies and adult parents who served as the nutritional gatekeeper in the household (ie, the family member responsible for at least half of the food planning and preparation). Potential participants were contacted from a volunteer participant database maintained at the Max Planck Institute for Human Development. Note that the preregistration stated that children between 6 and 10 years would be recruited. Due to a misunderstanding, we also recruited 1 child aged 11 years. We cannot think of any reason that this incident should affect the results and conclusions.

All participants underwent 2 mealtime conditions (control or regular and intervention or longer), but they were randomized to the condition they would complete first. The order of the 2 conditions was randomized using a block randomization procedure (AB/BA design), and the interval between conditions was 1 to 3 weeks.

### Intervention

First, parents completed an online preassessment together with their children at home. Second, they were invited to the laboratory for 2 free evening meals, which took place under different conditions. In the regular condition, each dyad was given the same amount of time to finish the meal as their reported regular mealtime duration (mean [SD] reported duration, 20.83 [7.46] minutes). In the longer condition, each dyad had 50% more time than their reported regular mealtime duration (on average, 10 minutes longer).

Participants were served a typical German evening meal of sliced bread, cold cuts of cheese and meat, and bite-sized pieces of fruits and vegetables. At the end of the meal, the table was cleared and participants were offered a dessert of chocolate pudding or fruit yogurt and cookies. Water and 1 sugar-sweetened beverage were provided throughout the meal. All foods and beverages served reflected the child’s preferences as reported in the online preassessment. Participants were instructed to not eat anything in the 2 hours before coming to the laboratory for the free meals. Before beginning their meal, participants were informed of the exact time the investigator would return to clear the table. For example, in the regular condition, if the family’s regular mealtime duration was 20 minutes and the meal started at 6 pm, participants were informed that the investigator would return at 6:20 pm. We deliberately avoided stating that the participants had 30 (vs 20) minutes available because we did not want to draw attention to the trial objective. Poststudy inquiry showed that no participants suspected that the trial was related to mealtime duration. Third, participants completed a postmeal questionnaire on satiety and perceived mealtime atmosphere.

### Trial Outcomes

Video cameras simultaneously recorded the meals from 2 angles: frontal view of the child and frontal view of the parent. The recordings were merged into 1 double-view movie and coded using Datavyu software, version 1.2.2 (Datavyu Team).^16^

Food consumption of the parent-child dyads was coded from the video recordings by independent coders using a standardized coding system. Key variables were the number of pieces of fruits and vegetables, the number of bread and cold cuts (ie, slices of bread, cheese, and cold cut meat; teaspoons of butter and sweet spreads), the amount of dessert (teaspoons of chocolate pudding or fruit yogurt; number of cookies), and the amount of water and sugar-sweetened beverages in milliliters (estimated to the nearest 100 mL). All participants were offered the same food and beverage categories (ie, fruits and vegetables, bread and cold cuts, dessert, and water and sugar-sweetened beverages) and serving sizes. Foods served were held constant within families across regular and longer conditions. The exact food type (eg, type of bread and type of fruit) reflected the child’s preferences (eg, favorite bread and top-3 preferred fruits) that were reported in the online preassessment and thus varied between families. Due to the natural variation in the size of fruits and vegetables, it was not possible to ensure that all pieces were uniform. The weight range was 10 to 14 g for cherry tomatoes and 6 to 10 g for grapes and tangerine segments. Other fruits and vegetables (eg, apples and cucumbers) were cut into pieces weighing 9 to 11 g. Variability in size likely balanced out across conditions.

In an independent pilot study (N = 10), negative communication was not observed within dyads (Mattea Dallacker, PhD, unpublished data, 2016). We therefore measured variability between the 2 conditions in self-rated mealtime atmosphere and observed the amount of interpersonal communication. Interpersonal communication was defined as positive or neutral verbal information exchange (eg, about interests or family life), including joking and commenting on feelings or emotions, and was coded from the video recordings using a standardized system (ABC Mealtime Coding System^17^). The proportion of interpersonal communication in milliseconds compared with total mealtime was calculated as an indicator of mealtime atmosphere.^17^

The number of bites taken per minute was coded from the video recordings (see Llewellyn et al^18^ for a similar procedure). Mean bites per minute were calculated by dividing the number of bites, counted in the same amount of time in both conditions (ie, regular mealtime duration), by the regular mealtime duration (in minutes). This calculation permitted us to compare the eating rate in the regular condition with the eating rate in the longer condition during the same time window.

Coding was conducted by 2 trained research assistants who were blinded to the trial objective. An independent rater coded a randomly selected 20% of the videos. Interrater reliability was high for food consumption (intraclass correlation coefficient, 0.964-0.997) and interpersonal communication (intraclass correlation coefficient, 0.934).

Demographic characteristics, family mealtime duration, and food preferences of the child were collected in the online preassessment. All participants were from Berlin, Germany; ethnicity was not assessed. Parents were asked to measure the duration of their next main family meal and to use this duration as a basis for estimating their regular mealtime duration. Children’s food preferences were measured using a 5-point Likert-type scale, which was adapted from Fildes and colleagues,^19^ that rated 40 food and drink items typically served at an evening meal in Germany, with 1 indicating *dislikes a lot* to 5 indicating *likes a lot*. The top-ranked items (with a score of at least 3 for *likes a bit*) were served at the laboratory meal.

After both evening meals at the laboratory, parents rated their satiety on a visual analog scale with the poles of *not hungry at all* and *extremely hungry*,^20^ and they rated the atmosphere of the meal on a 5-point Likert scale of 1 indicating *very negative* to 5 indicating *very positive*.^21^ Children rated their satiety using a picture rating scale.^22^

### Data Preparation

To compare families whose mealtime durations differed, we converted the mealtime from minutes to percentages, with 0% to 100% of mealtime referring to the regular mealtime duration (in both regular and longer conditions) and 100% to 150% of mealtime referring to the extra time (longer condition only). Start and end times of interpersonal communication, start time of each bite, and each food consumed were exported from the Datavyu platform.

### Statistical Analysis

One- or 2-sided, paired *t* tests were performed to compare food consumption in the 2 conditions. A longitudinal multilevel analysis (random slopes and fixed intercept) was conducted to explore consumption dynamics. First, we tested for both conditions and separately for fruits and vegetables whether a linear or a logarithmic curve better described cumulative consumption over time. A linear pattern would suggest that the more time children are given for eating, the more fruits and vegetables they consume at a meal, whereas a logarithmic pattern would suggest that children usually stop eating after a certain amount of time. Percentage of mealtime served as a level 1 variable, with the cumulative number of pieces of fruits and vegetables consumed as the dependent variable.

Second, we included condition as a level 2 variable in the better-fitting model. Using the cross-level interaction of the 2 independent variables, we could explore whether the increase in fruits and vegetables (primary outcomes) consumed by the end of the regular meal duration differed between conditions. We further tested for differences between conditions in the secondary outcomes: amount of interpersonal communication and eating rates (using paired *t* test) and self-rated atmosphere and satiety (using Wilcoxon signed rank test). In both groups, bites per minute in the regular mealtime duration (ie, 0%-100%) served as the dependent variable.

The statistical tests used are generally robust against violations of normal distributions in a sample with more than 30 participants. Nevertheless, we checked for violation of normal distribution using the Shapiro-Wilk test. In case of violation, we reran the analyses using nonparametric tests. In all cases, the results of the parametric and nonparametric test results were equivalent. All participants provided complete data. *P* < .05 indicated significance for all statistical tests. For 2-sided tests, we reported the upper and lower bounds of 95% CIs; for 1-sided tests, only 1 bound was reported, with the other bound being infinity. Statistical analyses of the full sample were conducted between June 2 and October 30, 2022, using R, version 3.2.3 (R Foundation for Statistical Computing).

## Results

Fifty parent-child dyads participated in the trial. Parents had a mean (range) age of 43 (28-55) years and included 36 mothers (72%) and 14 fathers (28%); no participants identified as nonbinary. Children had a mean (range) age of 8 (6-11) years and included 25 girls (50%) and 25 boys (50%). Of the parents, 41 (82%) completed academic-track secondary education level. Sample size was calculated based on a meta-analysis on mealtime duration and children’s nutritional health^7^ using G*Power.^23^ With an assumed effect size of Cohen *d* = 0.4 (power = 0.85; α = .05), a total sample of 47 dyads was required.

### Primary Outcomes

#### Fruit and Vegetable Consumption

**Figure 2.**
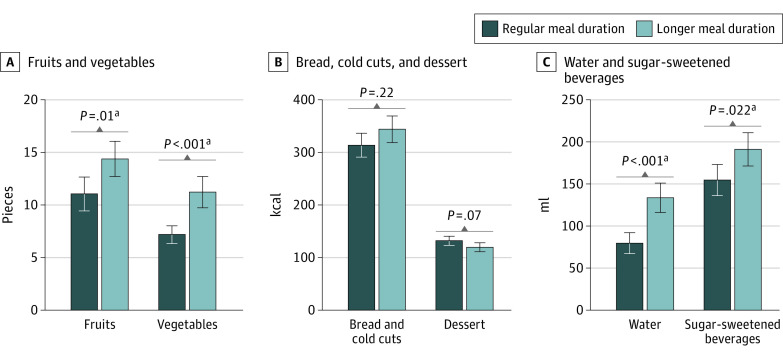
Children’s Food Consumption by Condition and Food or Beverage Category Fruits were approximately 10 g per piece (6-10 g for grapes and tangerine segments; 10-14 g for cherry tomatoes; and 9-11 g per cut piece of apple, banana, carrot, or cucumber). Cold cuts include cheese, cold cut meats, butter, and sweet spreads. Error bars represent the SEs of the means. ^a^The significant difference between regular and longer mealtime duration conditions.

As we hypothesized, children ate significantly more pieces of fruits (*t*_49_ = 2.36, *P* = .01; mean difference [MD], 3.32 [95% CI, 0.96 to ∞]; Cohen *d* = 0.33) and vegetables (*t*_49_ = 3.66, *P* < .001; MD, 4.05 [95% CI, 2.19 to ∞]; Cohen *d* = 0.52) in the longer than in the regular mealtime duration condition. The consumption of bread and cold cuts in kilocalories did not differ significantly between conditions (*t*_49_ = 1.25, *P* = .22; MD, 30.4 [95% CI, −18.28 to 79.08]; Cohen *d* = 0.18), but children drank significantly more milliliters of water (*t*_49_ = 3.70, *P* < .001; MD, 54.2 [95% CI, 24.73-83.67]; Cohen *d* = 0.52) and sugar-sweetened beverages (*t*_49_ = 2.37, *P* = .02; MD, 36.5 [95% CI, 5.53-67.47]; Cohen *d* = 0.34) in the longer condition (Figure 2; eTable 2 in Supplement 2). We found similar results in parents (eTable 1 in Supplement 2).

To address potential order effects of the longer condition, we descriptively examined whether the patterns of results replicated across both orders. Results were replicated for all primary outcomes. Given the high educational level among parents in the sample, we reran the analyses and included only those children whose parents had not completed academic-track secondary education. We found equivalent results with respect to direction and effect size.

#### Consumption Dynamics 

To explore consumption dynamics over time, we specified a linear and a logarithmic mixed model for the regular and longer conditions. For both conditions, the linear model showed a better fit for fruits (longer condition: Akaike information criterion [AIC], 37 420 vs 41 888; regular condition: AIC, 21 279 vs 25 554) and vegetables (longer condition: AIC, 29 577 vs 41 888; regular condition: AIC, 16 706 vs 19 856). eFigures 1 and 2 in Supplement 2 showed the cumulative distribution of fruits and vegetables consumed over time for each condition and for each child observed. To investigate this finding further, we included condition as a level 2 variable in the better-fitting (linear) model. For vegetables, the cross-level interaction between percentage of mealtime and condition was significantly different from 0 and there was an interaction for the longer condition (*b* = 0.01; *P* = .001). This finding means that children had already eaten more pieces of vegetables by the time their regular mealtime was over in the longer condition (ie, 100% of 150% mealtime duration) compared with the regular condition (100% of 100% mealtime duration). For fruits, a significant cross-level interaction emerged ((*b* =  −0.01; *P* < .001), indicating that children had eaten fewer pieces of fruit by the end of their regular mealtime in the longer vs regular condition. Because more fruit was eaten in the longer condition, this result suggests that the additional fruit was consumed during the extra time.

### Secondary Outcomes

The proportion of time spent engaged in positive interpersonal communication did not differ significantly between the regular and the longer conditions (*t*_49_ = 1.36, *P* = .09; MD, 3.2 [95% CI, −0.75 to ∞). Likewise, there was no significant difference in self-rated atmosphere between the 2 conditions (*V* [Wilcoxon signed rank test] = 126.5, *P* < .71; 95% CI, −1.00 to 0.00).

Children’s eating rate (bites per minute) was significantly lower in the longer than in the regular condition (*t*_49_ = −7.60, *P* < .001; MD, −0.72 [95% CI, −0.56 to ∞]; Cohen *d* = 1.08). Children reported significantly greater satiety in the longer than in the regular condition (*V* = 36.5, *P* < .001; 95% CI, 2.00 to ∞). However, consumption of dessert in kilocalories was not significantly lower in the longer condition (*t*_49_ = −1.47, *P* = .07; MD, −12.3 [95% CI, 1.69 to ∞]; Cohen *d* = 0.21).

## Discussion

This randomized clinical trial found that children consumed significantly more fruits and vegetables when family meals lasted 10 minutes longer, on average. The 7 additional pieces of fruits and vegetables (on average) corresponded to approximately 1 portion or 100 g (eg, 1 medium apple).^24^ This outcome has practical importance for public health because 1 additional daily portion reduces the risk of cardiometabolic disease by 6% to 7%.^25^ Moreover, this outcome was specific to fruits and vegetables; children did not eat significantly more of the other foods on offer. This finding is in line with results of cross-sectional studies showing that longer family meals are associated with better diet quality in children^7^ as well as with observational studies^26^ and 1 randomized clinical trial^27^ in the school context. That trial^27^ found that extending school lunch time increased fruit and vegetable intake.

Higher intake of fruits and vegetables during longer meals cannot be explained by longer exposure to food alone; otherwise, an increased intake of bread and cold cuts would have occurred. One possible explanation is that the fruits and vegetables were cut into bite-sized pieces, making them convenient to eat. Previous studies found that longer exposure to accessible foods increased the intake of these foods.^28,29^ Inconvenience or friction^30^ may explain why children did not consume more of the main components, such as bread or cheese, during longer meals; grabbing a bite-sized piece of fruit seemed more convenient than topping a slice of bread with cheese. Explorative analyses showed that the longer the meal lasted, the more fruits and vegetables the children ate and that more vegetables were eaten from the start, whereas the additional fruit was consumed during the extra time.

Additionally, longer family meals were associated with a slower eating rate, increased satiety, and a lower risk of obesity in children^31^ potentially because increased satiety played a role in reduced snacking between meals.^32^ We did not find a more positive mealtime atmosphere during the longer condition possibly because the laboratory setting led to more socially desirable communication, resulting in a ceiling effect.^33^

### Strengths and Limitations

The within-dyad manipulation design using video observation permitted causal inferences to be drawn. Despite this major strength, findings from the laboratory setting cannot simply be generalized to natural eating environments. Other limitations are that video observations can increase socially desirable behaviors,^34^ the sample had limited ethnic and socioeconomic diversity, and it remains unclear whether the effect of the intervention can be maintained over time. Some limitations were mitigated by the use of a within-dyad design. Comparing dyads with themselves makes it possible to control for situational factors (eg, video observation in both regular and longer conditions) and sample characteristics. Nevertheless, further studies should examine the effects of longer mealtime duration in more diverse samples and across longer time frames.

## Conclusions 

Results of this randomized clinical trial suggest that increasing family mealtime duration by approximately 10 minutes can improve children’s diets and eating behavior. How can families establish new routines with longer mealtimes? Possibilities include focusing on the mealtime that is most likely to succeed (ie, not breakfast when everyone is in a rush), accommodating children’s preferences (eg, playing music they have chosen in the background), and setting transparent rules (eg, everyone stays at the table for a certain time). These strategies may not always work; habit change takes effort but the necessary competences can be fostered.^35^ The effect of family meal duration on children’s intake of fruits and vegetables requires the availability of fruits and vegetables on the table. If the effects of this simple, inexpensive, and low-threshold intervention prove stable over time, it could contribute to addressing a major public health problem.

## Notes

Supplement 1.
Trial Protocol

Supplement 2. **eTable 1.** Food and Beverages Consumed by Adults (N = 50) **eTable 2.** Food and Beverages Consumed by Children (N = 50) **eFigure 1.** Cumulated Pieces of Fruits Consumed by Each Child in the Two Conditions **eFigure 2.** Cumulated Pieces of Vegetables Consumed by Each Child in the Two Conditions

Supplement 3.
Data Sharing Statement
