# Supplementary material for: Effect of Longer Family Meals on Children’s Fruit and Vegetable Intake: A Randomized Clinical Trial
Source: JAMA Netw Open. 2023 Apr 3;6(4):e236331. doi: 10.1001/jamanetworkopen.2023.6331 (PMC10071335; doi:10.1001/jamanetworkopen.2023.6331)
Supplement: Supplement 1. — Trial Protocol [file jamanetwopen-e236331-s001.pdf]

Study protocol

Study Title:

Effect of Longer Family Meals on Children's Fruit and Vegetable Intake: A Randomized Trial

Summary

The goal of this study is to test whether a longer meal duration could improve the diet quality of children. To answer this question, we want to take an experimental approach by implementing a longer family meal duration to examine differences in children's eating behavior. The family dinner within a laboratory setting will be video-taped and the main outcome is children's fruit and vegetables consumption.

Detailed Description

Parent-child pairs have two meals in a laboratory setting. The study design is a within-subject design: In the control condition they have as much time as usual. In the intervention condition they have 50% more time than usual.

Condition's order is counter balanced. The lab dinner reflects a typical German dinner which consists of bread, cheese, cold meat and fruits and vegetables. Additionally, a dessert is served after the main meal. The foods served reflect food preferences of the child. All dinners are video-taped. Key outcome variables are consumption of fruits and vegetables, dessert, eating rate and amount of time engaged in positive and negative social interaction.

Methods: Intervention, Participants, Outcome

Study type: Within subject randomized trial.

Time frame: November 2016 – May 2017

Intervention: Two conditions: Usual family meal duration versus 50%-increased duration. The family's usual meal duration will be assessed beforehand (online preassessment at home). Before each lab dinner starts, parent and child are told at which time the experimenter comes back to clear the table (e.g., if the family's usual meal duration was 20 minutes, and the lab dinner started at 6 p.m., then, in the experimental condition, family dyads are told that the experimenter comes back at 6:30; in the control condition at 6:20).

Eligibility Criteria

- Parents must be the household's nutritional gatekeeper
- Parents must have at least one child between the age of 6-11
- Participants should not follow a special diet (e.g., no food allergies)
- Consent to participate and be video-taped during the lab dinners

Randomization

- Each parent-child dyad will have two lab dinners, one dinner at usual meal duration, one dinner that takes 50% longer (2 week washing-out phase in between)
- Control and intervention condition are presented in randomized order

- Randomization technique: Block randomization

Recruitment

Participants are recruited through a participant data base of the lab of the Max Planck Institute for Human Development. Importantly, these participants have not participated in a food or health-related experiment at the institute before. Recruitment and allocation procedure are conducted by the lab manager of the Max Planck Institute for Human development and well as two trained research assistants.

Set-up laboratory meal

All laboratory dinners are recorded with two video cameras (GoPro Hero 4, black edition). The cameras captured the dinners from two different angles (frontal view of the child and frontal view of the parent). The dinner room is set up with plants, pictures on the wall, and cozy lighting to resemble a family dining room. The participants eat foods freely from the options provided on the dinner table, which reflects a typical German dinner: All lab dinners consist of sliced bread, cheese, and cold meat, butter, sweet spreads (e.g., marmalade or hazelnut crème) and pieces of fruits and vegetables of equal size. Beverages provided include water and one sugar sweetened beverage. After the main meal, a dessert is served, consisting of pudding or fruit yogurt and cookies. Importantly, the types of food and beverages served at the lab dinner (e.g., type of vegetable, type of cheese, type of sugar sweetened beverage, type of dessert) matches the child's individual food preferences, as assessed in the preassessment. The family is instructed to not eat anything during the two hours before coming to the institute.

Procedure

Before the lab sessions starts, parents are asked to fill in an online questionnaire at home together with their child (Preassessment). This is to receive information on the child's food preferences and the family's usual meal duration. This information is used to set up the lab dinner. After filling out the online questionnaire, parents are contacted in order to make two appointments for the lab dinners at the Max Planck Institute for Human Development. Directly after each lab session, parents and children complete a short questionnaire about satiety and experiences during the lab meal.

Figure 1 Study procedure with time frame

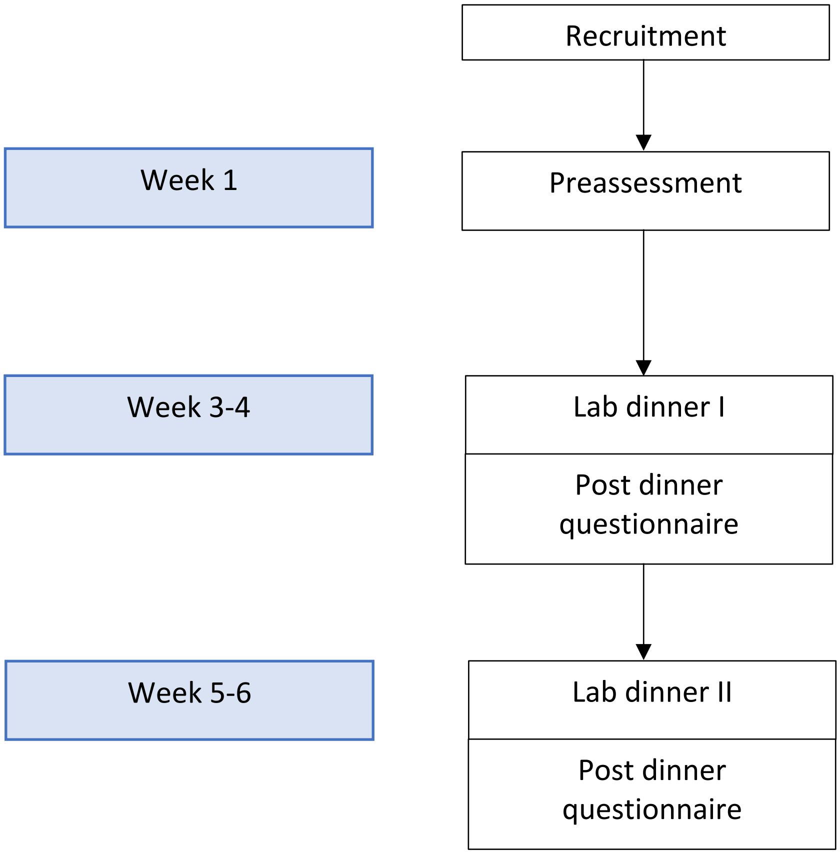

Video coding

The videos are clustered into one double-view movie merging the two recordings and are coded with the coding software datavyu. The coding is conducted by two trained research assistants who are blind to the study purpose. Research assistants participated in several training sessions. They received information on judgmental biases, family meals and eating behavior in children. They are introduced to the coding manuals, familiarize with the coding software datavyu and practice coding with videos from the pilot study.

Coding food consumption

We use either standardized measuring spoons and glasses or individual pieces to allow all observers to visually estimate the portions. Food intake during the lab dinner is coded as follow:

- Number of pieces of fruits
- Number of pieces of vegetables
- Number of slices of bread (0,5 steps)
- Number of slices of cheese (0,5 steps)
- Number of slices of cold meat (0,5 steps)
- Number of teaspoons of butter
- Number of teaspoons of sweet spread
- Number of teaspoons of dessert (yoghurt or pudding)
- Number of cookies
- Water in ml (participants drink from transparent glass with marks at 100 ml, 200ml, 300ml)

- Sugar sweetened beverages in ml (participants drink from transparent glass with marks at 100 ml, 200ml, 300ml)

The kilocalories for each piece of food (e.g. one slice of bread) or for a teaspoon of food (e.g. butter or dessert) are calculated based on their weight and the information from the respective nutrition label.

Coding interpersonal communication

Interpersonal communication is defined as positive or neutral verbal information exchange (e.g., about facts, beliefs, interests, or family life), and includes joking or comments on feelings or emotive actions. A communication starts the second a participant begins to talk and ends the second of their last word before silence or when someone else begins a conversation. The proportion of interpersonal communication in milliseconds compared to total dinner time is calculated and serves as an indirect indicator of the mealtime atmosphere.

See ABC coding manual from Family Research Lab, Syracuse University, (Fiese, Winter, & Botti, 2001) for more information.

Coding eating rate

Eating rate is measured as bites taken per minute coded from the videotapes following the procedure described by Llewellyn et al. (2008).

- A bite is counted whenever it is clearly visible that the child bit a piece of a food.
- Nibbling is counted as a bite only when an amount the size of a bite is ingested by several consecutive nibbles
- Isolated nibbles are not counted

- Amounts smaller than what is defined to qualify as a bite count as a bite only when they are consumed multiple times in a row and the summed-up amount is estimated to equal a bite
- Eating rate is calculated by dividing the total number of bites within a time unit by its duration (see data preparation for more details on time units).
- Sucking is not counted as a bite

Preassessment

Before the first lab dinner, parents are asked to fill out an online survey at home together with their child. Variables include:

- Demographics (age of the child and the parent, education of the parent)
- the family's usual meal duration (parents are asked to measure the duration of the family's next main meal and to use this as a basis to estimate the usual family meal duration). This information is used to determine the meal duration for the lab sessions.
- **Child's food preferences:** Food preferences of the child are measured using a food questionnaire adapted from Fildes and colleagues. The scale contained 40 food and drink items chosen to cover the range of foods typically served for dinner in Germany (measured 5-point- Likert-type scale, anchored between "dislikes a lot" and "likes a lot"). Based on the results of the preference scale, the top ranked (at least scale point 3, "likes a bit") foods and drinks are chosen for the lab dinner. We add smileys to the scale in order to make it easier for the child to rate their liking of the food:

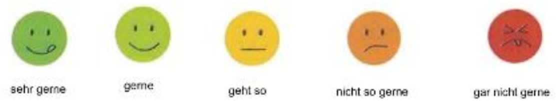

Post-dinner questionnaire

- **Satiety of the child.** A picture rating scale adapted from Bennett & Blissett (2014) is used to rate their hunger and satiety

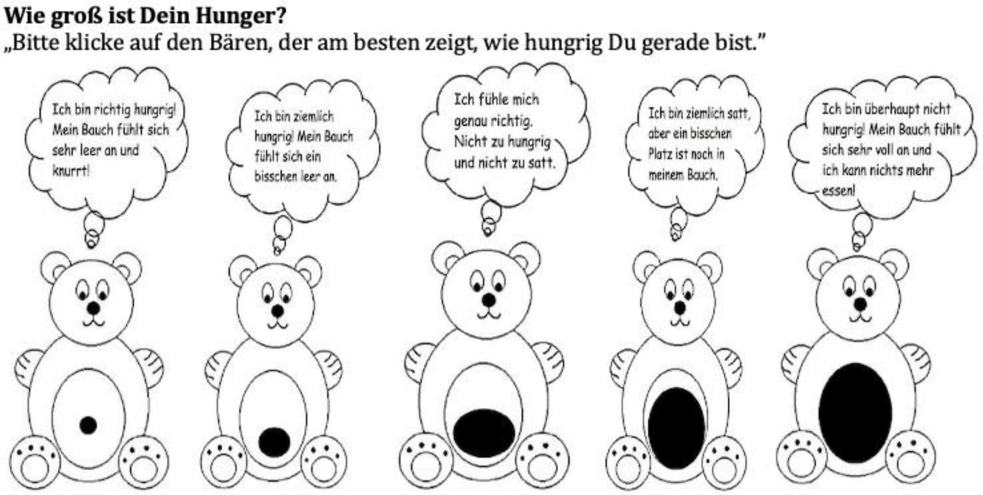

- **Satiety of the parent.** Sensation of hunger and thirst on a visual analogue scale with the opposites "not hungry at all" and "extremely hungry' (Flint et al., 2000).

- **Mealtime atmosphere.** Parents were asked to rate the atmosphere of the lab dinner on a 5-point Likert scale ranging from very negative to very positive (Knobl et al., 2022).

Statistical Analyses

Data preparation

Mealtime is converted from minutes to percent to compare children with different usual duration times. So, the videos are divided up into time units: 0%- 100% of mealtime refers to the usual meal duration (in both, the control and experimental condition), 100%-150% of mealtime refers to additional time (in the experimental condition only). Start and end time of social interactions,

start time of each bite, and each food consumed are exported from datavyu. The frequency and duration of social interactions are calculated in Excel.

Sample size

Sample size is calculated based on a meta-analysis on meal duration and children's nutritional health, using G*Power software. With the assumed effect size of d = 0.4 (with power =.85, alpha =.05), a total sample of 47 families is required.

Variables

- Mealtime (usual vs. longer meal duration) serves as independent variable.
- Food consumption (continuous variable) is the main outcome of interest and serves as dependent variable.
- Further outcome variables: satiety (ordinal variable), perceived mealtime atmosphere (ordinal variable), interpersonal communication (continuous variable), eating rate (continuous variable)

Descriptive statistics include

- Age parent
- Age child
- Gender parent
- Gender child
- Educational level of the parent

Statistical analyses

- Paired t-test to compare food consumption between the usual meal duration condition and longer meal duration condition
- longitudinal multilevel approach (random slopes, fixed intercept) to explore consumption dynamics of fruits and vegetables over time (i.e., when additional fruits and vegetables in the longer duration condition where eaten)
    - test whether a linear or logarithmic curve better describes consumption over the time of the meal; percent of mealtime as predictor on Level 1, cumulated pieces of fruits and vegetables as dependent variable.
    - take better-fitting model; include condition as a Level 2 predictor; use cross-level interaction of the two predictors to explore whether the increase in consumed pieces differs between groups up to the meal duration of 100%
- paired t-test to test for differences interpersonal communication between meals at usual length and prolonged meals

- Wilcoxon signed rank test to test for differences in self-rated atmosphere between meals at usual length and prolonged meals
- Wilcoxon signed rank test to test for differences in satiety between meals at usual length and prolonged meals
- paired t-tests to test for differences in eating rate between the usual meal duration and longer meal duration condition; dependent variable: bites per minute within the first time unit (i.e. usual meal duration = 100%). We expect the overall eating rate (measured in bites per minute) to decrease in the longer meal duration condition, because it is very likely that children do not eat 50% more when they have 50% more time, which

inevitably leads to fewer bites per minute. However, we are interested in the question if children *start* eating at a slower rate when they have more time to eat. Consequently, we compare the first- 100%- time units of both conditions only to test the mechanism of eating rate.

Statistical analyses are performed in R 3.2.3

References

Fiese BH, Winter MA, Botti JC. The ABC's of Family Mealtimes: Observational Lessons for Promoting Healthy Outcomes for Children with Persistent Asthma. Child Dev. 2011; 82(1):133–45.

Llewellyn, CH., van Jaarsveld, CHM., Boniface, D., Carnell, S., & Wardle, J. Eating rate is a heritable phenotype related to weight in children. *Am J Clin Nutr*. 2008; 88, 1560–1566. https://doi.org/10.3945/ajcn.2008.26175

Bennett C, Blissett J. Measuring hunger and satiety in primary school children. Validation of a new picture rating scale. *Appetite* 2014;78:40–8. doi:10.1016/j.appet.2014.03.011

Flint A, Raben A, Blundell JE, et al. Reproducibility, power and validity of visual analogue scales in assessment of appetite sensations in single test meal studies. *Int J Obes Relat Metab Disord J Int Assoc Study Obes* 2000;24:38–48. doi:10.1038/sj.ijo.0801083

Knobl V, Dallacker M, Hertwig R, *et al.* Happy and healthy: How family mealtime routines relate to child nutritional health. *Appetite* 2022;171:105939. doi:10.1016/j.appet.2022.105939
