# Supplementary material for: Effect of Longer Family Meals on Children’s Fruit and Vegetable Intake: A Randomized Clinical Trial
Source: JAMA Netw Open. 2023 Apr 3;6(4):e236331. doi: 10.1001/jamanetworkopen.2023.6331 (PMC10071335; doi:10.1001/jamanetworkopen.2023.6331)
Supplement: Supplement 2. — eTable 1. Food and Beverages Consumed by Adults (N = 50) eTable 2. Food and Beverages Consumed by Children (N = 50) eFigure 1. Cumulated Pieces of Fruits Consumed by Each Child in the Two Conditions eFigure 2. Cumulated Pieces of Vegetables Consumed by Each Child in the Two Conditions [file jamanetwopen-e236331-s002.pdf]

Supplemental Online Content

Dallacker M, Knobl V, Hertwig R, Mata J. Effect of longer family meals on children's fruit and vegetable intake: a randomized clinical trial. *JAMA Netw Open*. 2023;6(4):e236331. doi:10.1001/jamanetworkopen.2023.6331

eTable 1. Food and Beverages Consumed by Adults (N = 50)

eTable 2. Food and Beverages Consumed by Children (N = 50)

eFigure 1. Cumulated Pieces of Fruits Consumed by Each Child in the Two Conditions

eFigure 2. Cumulated Pieces of Vegetables Consumed by Each Child in the Two Conditions

This supplemental material has been provided by the authors to give readers additional information about their work.

© 2023 Dallacker M et al. *JAMA Network Open.*

eTable 1. Food and Beverages Consumed by Adults (N = 50)

Food item	Regular meal duration M (SD)	Longer meal duration M (SD)	Mean difference (95% CI)	*P*	Cohen's *d*
Fruits (pieces)	15.04 (12.01)	19.34 (12.38)	4.3 (2.02)	0.001	0.45
Vegetables (pieces)	17.88 (11.22)	20.7 (10.3)	2.82 (0.53)	0.022	0.29
Bread and cold cuts* (kcal)	347.1 (140.28)	396.73 (157.99)	49.63 (5.75 – 93.5)	0.028	0.32
Sugar-sweetened beverages (ml)	81.2 (103.97)	103.5 (135.71)	22.3(-1.32 – 45.92)	0.064	0.27
Water (ml)	230.2 (155.86)	283 (164.02)	52.8 (11.08 – 94.52)	0.014	0.36
Dessert (kcal)	87.1 (72.33)	83.7 (71.71)	-3.4 (6.65)	0.287	0.08

*Cold cuts include cheese, cold meat, butter, and sweet spreads.

© 2023 Dallacker M et al. *JAMA Network Open.*

eTable 2. Food and Beverages Consumed by Children (N = 50)

Food item	Regular meal duration M (SD)	Longer meal duration M (SD)	Mean difference (95% CI)	*P*	Cohen's *d*
Fruits (pieces)	11.06 (11.33)	14.38 (11.86)	3.32 (0.96)	0.011	0.33
Vegetables (pieces)	7.18 (6.03)	11.23 (10.51)	4.05 (2.19)	<0.001	0.52
Bread and cold cuts* (kcal)	313.53 (159.62)	343.93 (178.82)	30.4 (-18.28 – 79.08)	0.216	0.18
Sugar-sweetened beverages (ml)	155.2 (129.19)	191.7 (140.57)	36.5 (5.53 – 67.47)	0.022	0.34
Water (ml)	80 (87.69)	134.2 (124.08)	54.2 (24.73 – 83.67)	<0.001	0.52
Dessert (kcal)	131.9 (60.11)	119.6 (60.71)	-12.3 (1.69)	0.073	0.21

*Cold cuts include cheese, cold meat, butter, and sweet spreads.

© 2023 Dallacker M et al. *JAMA Network Open.*

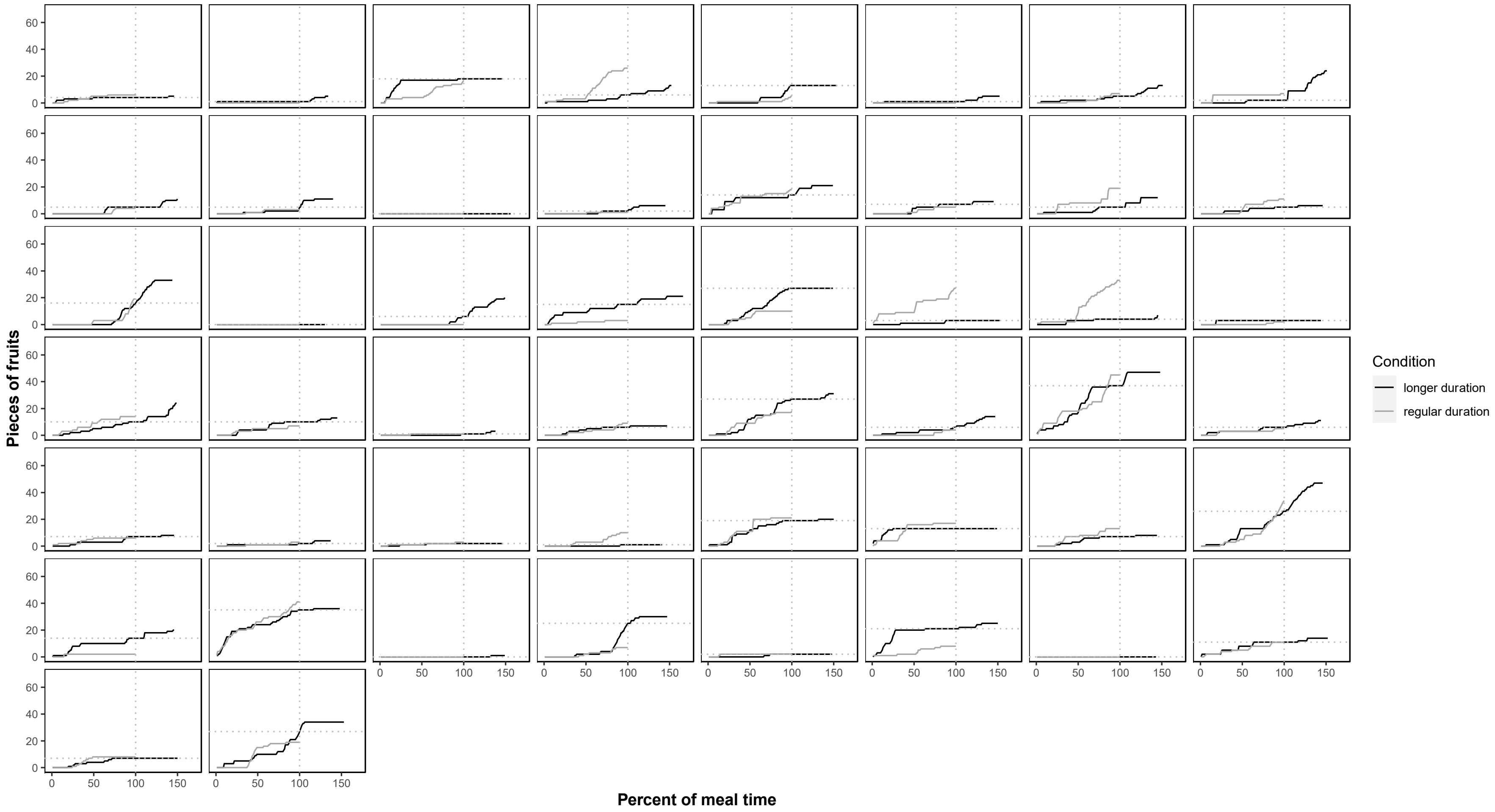

eFigure 1. Cumulated Pieces of Fruits Consumed by Each Child in the Two Conditions

Meal time on the X axis in percent with the usual mealtime duration as a baseline with up to 100% and the longer mealtime duration up to 150%; N = 50.

© 2023 Dallacker M et al. *JAMA Network Open.*

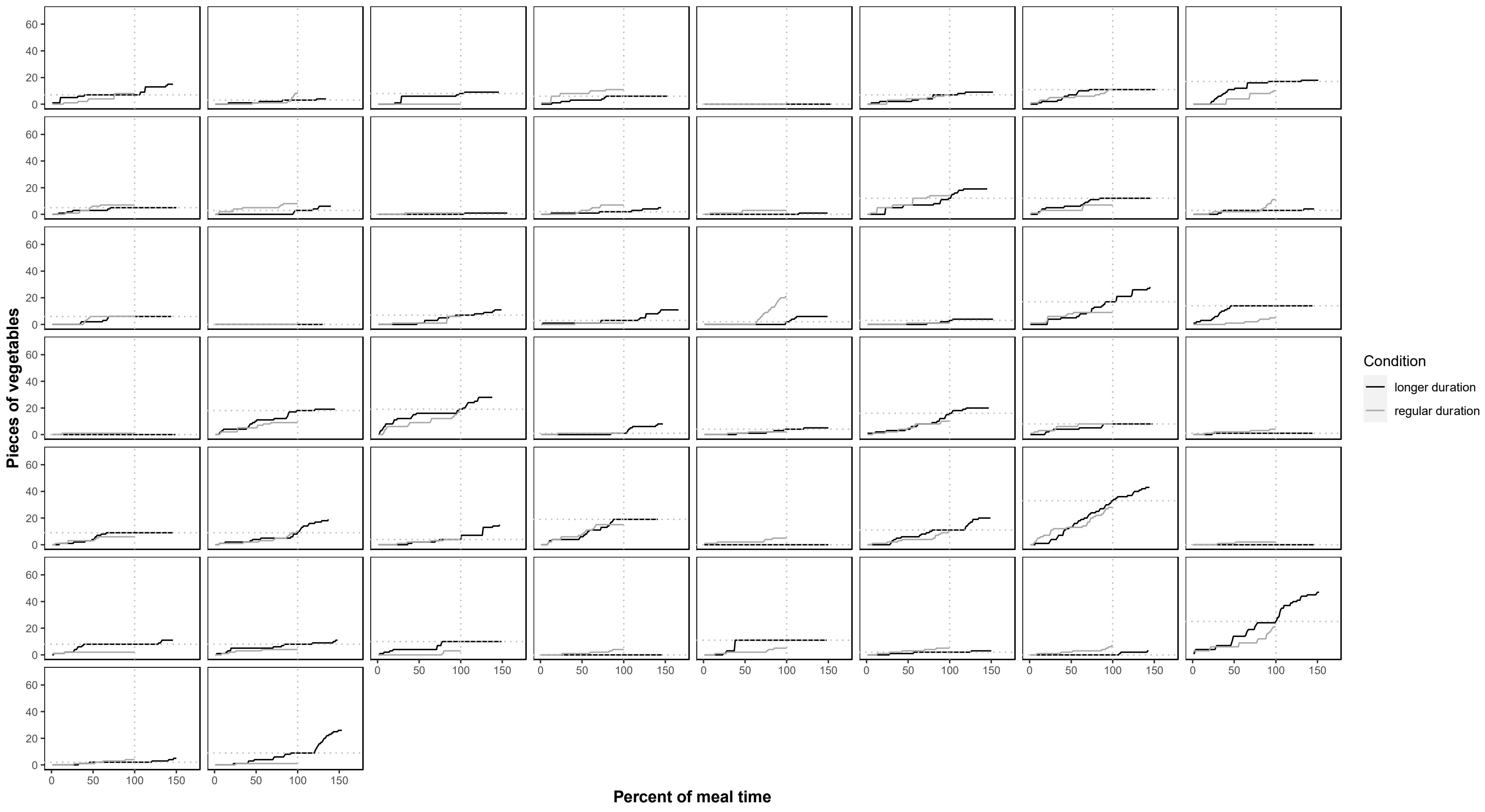

eFigure 2. Cumulated Pieces of Vegetables Consumed by Each Child in the Two Conditions

Meal time on the X axis in percent with the usual mealtime duration as a baseline with up to 100% and the longer mealtime duration up to 150%; N = 50.

© 2023 Dallacker M et al. *JAMA Network Open.*
