# Supplementary material for: Effect of Longer Family Meals on Children’s Fruit and Vegetable Intake: A Randomized Clinical Trial
Source: JAMA Netw Open. 2023 Apr 3;6(4):e236331. doi: 10.1001/jamanetworkopen.2023.6331 (PMC10071335; doi:10.1001/jamanetworkopen.2023.6331)
Supplement: Supplement 3. — Data Sharing Statement [file jamanetwopen-e236331-s003.pdf]

Data Sharing Statement

Dallacker. Effect of Longer Family Meals on Children's Fruit and Vegetable Intake. *JAMA Netw Open*. Published April 03, 2023. doi:10.1001/jamanetworkopen.2023.6331

Data
Data available: Yes
Data types: Deidentified participant data
How to access data: Data are available on the Open Science Framework under the link: https://osf.io/3c8qa/?view_only=88aa4049f0354db6a3bfdeb34cb01fc0
When available: With publication

Supporting Documents
Document types: Statistical/analytic code
How to access documents: The statistical code is available on the Open Science Framework under the link: https://osf.io/3c8qa/?view_only=88aa4049f0354db6a3bfdeb34cb01fc0
When available: With publication

Additional Information
Who can access the data: anyone with access to the Open Science Framework
Types of analyses: for any purpose
Mechanisms of data availability: without investigator support
